# Coffee

**Published:** 1891-04

**Authors:** 


### Coffee.

After years of extended observation and pronounced personal experience I feel justified in announcing:

1. The world has in the infusion of coffee one of its most valuable beverages.

2. As a prompt diffusible stimulant, either by the stomach or by injection into the rectum, it is in all cases of shock preferable to alcohol.

3. It is antagonistic to malaria, and specially destructive to the typhoid bacillus and cholera germ, and for this reason it is an admirable remedial agent in these conditions both as a direct stimulant, an antiseptic and an encourager of elimination.

4. One of its chief advantages in health and disease is in the fact that it aids in the securement of that psychical satisfaction which is conducive to hope, comfort, good digestion, great power of resistance and rapid recuperation.

5. In season, it supports, tides over dangers, helps the appropriative powers of the system, whips up the flagging energies, enhances the endurance, but is in no sense a food, and for these reasons and many others, it should be used temperately, as should all of nature's benign gifts.

6. In excess it is even more dangerous than alcohol for it is not like the latter, a nutrient, nor is the effect of its excessive use so apparent or unrespectable.—*I. N. Love, M.D., St. Louis, Missouri.*

---

DEFECTIVE TEETH. To briefly recapitulate, teeth whose structure has been impaired during their development may be divided into two classes, according to their cause: 1st the teeth of congenital syphilis, which are usually unmistakable; 2nd, teeth whose defective structure owes its origin to either stomatitis or any of the eruptive fevers. In this class it is quite impossible to tell which disease has been the cause without inquiring into the history of the case, and even then it is not always possible to make up one's mind. W. S. HOLFORD, in the *Dental Record.*

